# Non-Invasive Rayleigh, Raman, and Chromium-Fluorescence Study of Phase Transitions: β-Alumina into γ-Alumina ‘Single’ Crystal and Then to α-Alumina

**DOI:** 10.3390/ma18204682

**Published:** 2025-10-12

**Authors:** Juliette Redonnet, Gulsu Simsek-Franci, Philippe Colomban

**Affiliations:** 1 Centre des Matériaux (CEMAT), Mines Paris PSL, Campus de l’Innovation, 21, allée des Marronniers, 78000 Versailles, France; juliette.redonnet@minesparis.psl.eu; 2 Saint-Gobain Research Province, 550 rue Alphonse Jauffret, 84300 Cavaillon, France; 3 Department of Materials Science and Nanotechnology Engineering, Faculty of Engineering, Istanbul Gedik University, Cumhuriyet Mah İlkbahar Sok. No:1 Kartal, 34876 Istanbul, Türkiye; gulsu.simsek@gedik.edu.tr; 4 MONARIS UMR8233, CNRS, Sorbonne Université, Campus P.-et-M. Curie, 4 Place Jussieu, 75005 Paris, France

**Keywords:** Raman spectroscopy, XRD, fluorescence, chromium, transition alumina, corundum, amorphous alumina, gamma alumina, theta alumina, beta alumina, single crystal, gel, glass

## Abstract

In many advanced materials production processes, the analysis must be non-invasive, rapid, and, if possible, *operando*. The Raman signal of the various forms of alumina, especially transition alumina, is very weak due to the highly ionic nature of the Al-O bond, which requires long exposure times that are incompatible with monitoring transitions. Here, we explore the use of the fluorescence signal of chromium, a natural impurity in alumina, and the Rayleigh wing to follow the crystallization process up to alpha alumina. To clarify the assignment of the fluorescence components, we compare the transformation of beta alumina single crystals into transition (gamma and theta) alumina and then into alpha alumina with the transformation of optically transparent alumina xerogel and glass, obtained by very slow hydrolysis-polycondensation of aluminum sec-butoxide, into alpha alumina. Vibrational modes are better resolved in thermally treated single crystals than in thermally treated xerogels. Measurements of the Rayleigh wing, the Boson peak, and the fluorescence signal are easier than those of vibrational modes for studying the evolution from amorphous to alpha alumina phases. The fluorescence spectra allow almost instantaneous (<1 s) quantitative control of the phases present.

## 1. Introduction

The very weak Raman signal of alpha alumina [1,2] and aluminates [3] is well established and attributed to the weakly covalent character of the Al-O bond. Monitoring the formation of alpha alumina from amorphous precursors using Raman spectroscopy is therefore difficult [4]. Furthermore, broad bands, which are more difficult to detect at low intensity, are expected for disordered and lacunar structures such as those of transition aluminas. Consequently, only a few optical spectroscopic data on transition aluminas exist in the literature, and most are rather old and of medium quality [2,5,6].

Because of its compact structure and high melting point, alpha alumina exhibits excellent mechanical properties at room temperature and retains these properties relatively well at elevated temperatures [7,8,9,10]. It is therefore used in different forms, films, matrices, or fibrous reinforcements (composites), to fabricate a wide range of thermo-structural and insulating materials [9,10,11,12,13], with some studies employing optical spectroscopy. Several types of alumina fibers are known. The first, developed in the 1980s and marketed as Saphikon^®^, is a large diameter (~130 µm) monocrystalline fiber, mainly intended for reinforcing metal matrices [11,13]. Later, small-diameter fibers (~10 µm) were produced by Mitsui Cy under the name Almax^®^, for use in fiber-reinforced vitreous or ceramic matrix composites [9,12]. These alumina materials are synthesized through chemical routes that first yield an amorphous phase.

In the search for non-invasive spectroscopic methods to establish a reliable and rapid procedure for monitoring the crystallization process of new alumina fibers during thermal treatments [14], we resumed earlier work carried out in 1988 [4] on the formation of transition aluminas, now with far more efficient instrumentation than was available 35 years ago. Since optical spectroscopies can be performed without contact with the sample, we employed both the Raman vibrational spectrum and the chromium fluorescence signal. Almost all alumina-based ceramic materials contain traces of chromium, which, under excitation with a green laser, produces a fluorescence signature. This fluorescence has been used to measure mechanical stresses in composites [9,10,11,12,13,15,16,17,18], in thermally grown oxide (TGO) layers forming on Al-containing alloys used in thermal-barrier-coated blades [5,6,7,8], and to measure hydrostatic pressures in diamond anvil cells using ruby spheres [19,20]. This method can also be used to monitor the stress state of fibers in a composite.

The chromium fluorescence spectrum shows distinct components in various spectral ranges, which are well characterized for the corundum phase (alpha alumina) but much less so for transition aluminas [5,6]. To address this, we took advantage of the possibility of obtaining quasi-single crystals of transition gamma alumina (with a spinel structure) by heat treatment of ammonium beta alumina single crystals [4,21]. This is, to our knowledge, the only method for synthesizing ordered “large” crystals of transition gamma alumina. Additional comparison with spectra recorded from thermally treated, optically transparent alumina xerogels and glasses [2,22] was also used to support the spectral assignments.

## 2. Materials and Methods

### 2.1. Samples

**Figure 1:**
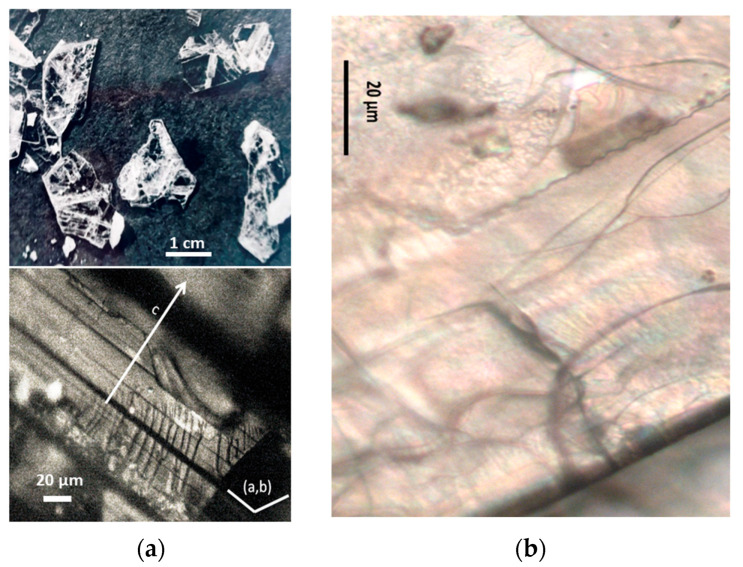
(**a**) Optical micrograph (×50 objective) of sodium beta alumina single crystalline platelets (**a**, **top**) and ammonium beta alumina single crystal transformed into gamma alumina (**a**, **bottom**, view of platelet section) after thermal treatment at 850 °C (The a, b, and c axes are the fundamental axes of the crystal unit cell). (**b**) Milky gamma alumina platelet with nucleation of alpha alumina phase (white spots) after thermal treatment at 1000 °C.

Non-stoichiometric beta and beta″ single crystals (thickness: 1 to 3 mm; size: 1 to 4 cm^2^) with compositions 11 Al_2_O_3_ 1.3 Na_2_O and 11 Al_2_O_3_ 1.6 Na_2_O, respectively, were prepared in the late 1970s by cooling from the liquid state (~2000 °C) using the self-crucible technique [3,21,23,24]. Heating was achieved with a powder mixture of high-purity sodium carbonate (Prolabo, Paris, France) and alpha alumina (Le Rubis des Alpes, Jarrie, France). Inductive 6 MHz coupling was first obtained with a graphite plate, manually oriented horizontally to maximize coupling. The copper crucible was water-cooled. At the contact with graphite, an Al_2_O_3_-Na_2_O liquid phase formed, progressively melting the entire powder mixture. Cooling to solidification took between 1 and 4 h, depending on the experiment, yielding a boule of ~1 kg of crystals. Crystal surface areas ranged from a few tens of mm^2^ to a few cm^2^, and thicknesses from 0.5 mm to several mm (Figure 1). The non-stoichiometry was compensated by extra interstitial oxygen atoms [21,23,24].

Similar sodium beta alumina single crystals were also extracted from commercially available Jargal H^®^ bricks (Saint-Gobain Refractory Division, Le Pontet, France), which are designed for use in glass industrial kilns (Table 1). The mechanism of non-stoichiometry compensation in Jargal^®^ crystals is not fully understood; impurities may contribute to compensation in addition to or instead of interstitial oxygen atoms.

Due to their layered structure, the crystals form platelets perpendicular to the c-axis of the unit cell (Figure 1). Crystals were ion-exchanged in molten NH_4_NO_3_ for a few hours and then washed three times with hot water (the exchange cycle was repeated twice), following the previously described procedure [3,21,23]. The non-stoichiometric beta crystals were then thermally treated at 500 °C for 4 h in air to obtain stoichiometric oxonium beta alumina (11 Al_2_O_3_ (OH_3_)_2_O). This occurs through elimination of the excess oxygen atoms located in the conducting plane by reaction with protons released from ammonium decomposition and air humidity [3,4,21,23]. Thus, stoichiometric oxonium beta alumina was obtained [3,21,23]. Subsequent thermal treatments between 800 and 1200 °C in air gradually transformed the material first into gamma alumina and then into alpha alumina [4,21,23]. During this process, the large variation in the c-axis unit-cell parameter caused the crystals to cleave and crack (Figure 1).

Optically clear alumina xerogel monoliths, prepared in the 1980s by very slow hydrolysis (over several months) of aluminum sec-butoxide in high-purity hexane (or hexane-acetone mixtures) according to Ref. [2], were also thermally treated at temperatures between 700 and 1800 °C in air (Table 1). Optical transparency was lost above 1100 °C due to nucleation of alpha alumina [2].

A synthetic MgAl_2_O_4_ single crystal wafer (MTI Corporation, Richmond, VA, USA) is used as reference.

### 2.2. Raman Spectroscopy

Raman and fluorescence spectra were obtained using a Labram HR800 spectrometer (HORIBA Scientific Jobin-Yvon, Palaiseau, France) excited with an Ar^+^ ion plasma laser (Innova I90C 6UV, Coherent Inc., Santa Clara, CA, USA). To achieve a wide spectral window, a 600 line/mm grating was used. A small confocal hole diameter (180 µm) was chosen to exploit the potential of Ultra-Low-Frequency (ULF) filters, which allow recording down to ~10 cm^−1^. The 514.5 nm exciting line was used, with a laser output power of 1 W, yielding ~50 mW at the sample.

Microscope objectives used included ×10 for selecting the area to be analyzed and long working distance (LWD) ×50 and ×100 for local analysis (Olympus, Tokyo, Japan). Counting times ranged from 30 min to several hours for spectra between −200 and 4000 cm^−1^, with 2–30 accumulations. For fluorescence signals recorded between 4000–8000 cm^−1^ (648–874.5 nm in absolute scale) and 14,400–14,800 cm^−1^ (18,741.9–18,760.7 nm), the counting time was 3 s with 10 accumulations per range. Convenient spectra could nevertheless be obtained in less than 0.5 s. Spectra collected in different spectral ranges were stitched using Labspec 6^®^ software, with a common overlap window of 200 cm^−1^. Wavenumber accuracy (±1 cm^−1^) was checked against the silicon wafer reference spectrum.

Due to the high laser power and the intrinsically weak signal of alumina phases, spectra were recorded with different objectives to evaluate possible optical contributions. The spectra presented here were recorded with the ×50 LWD Olympus objective, which showed no parasitic contributions (some other objectives produced a weak, broad band around 850 cm^−1^).

### 2.3. X-Ray Diffraction

Gently crushed and strongly crushed crystals were deposited on a low-background single-crystal silicon sample holder with grease and analyzed using a Bruker D8 DISCOVER diffractometer with a Bragg-Brentano Ɵ-Ɵ set-up equipped with a Cu anti-cathode. Scans were performed over the 2θ range of 19° to 100°, with sample rotation applied to ensure optimal irradiation of the entire sample surface. Data were collected with a step size of 0.03° and a counting time of 24s per step necessary to ensure detection of transition alumina.

Due to the platelet form of single crystals, even after careful grinding, the conservation of the anisotropic gain shape induces a preferential orientation, and the 00l peak of beta alumina residues is especially enhanced.

## 3. Results

### 3.1. Raman Signatures of Thermally Treated Beta Alumina

The term “beta alumina” (and likewise beta″ and beta‴) was originally a misnomer: early studies of the Al_2_O_3_-Na_2_O phase diagram [25] identified these phases as alumina, although they are actually aluminates containing sodium, potassium, or lithium depending on the direct synthesis conditions. The ions can be exchanged with many other M^+^ or M^2+^ ions [21,23].

In the 1970s, beta and beta″ aluminas attracted great interest as potential solid electrolytes for advanced sodium batteries and H_2_ sensors [21], and there has been a strong revival of interest in recent years.

Beta alumina (hexagonal symmetry, P6_3_/mmc space group) and beta″ alumina (R$\bar{3}$m symmetry) [3,24]) consist of blocks with a spinel structure (ABC oxygen packing) stacked along the c-axis, separated by a loosely packed layer (the a,b plane) containing sodium or ammonium ions (mobile) and oxygen ions that form Al-O-Al-bridges with the spinel blocks. Stoichiometry varies: the stoichiometric beta form has one conducting ion and one bridging oxygen ion per conducting plane, while non-stoichiometric forms have 1.3 or 1.6 mobile ions and bridges per plane, depending on the synthesis [3,21,23,24]. The stoichiometry of the beta″ form is 1.6 [23,24]. In beta alumina, the conducting plan is a mirror plane, but not in beta″ alumina. Excess oxygen atoms in the conducting plane induce defects in the spinel block to maintain charge compensation [23,24].

The IR, Raman, and neutron vibrational spectra of beta and beta″ aluminas were extensively studied in the 1980s [3,21,23,26,27,28,29,30,31,32,33,34,35].

For comparison, gamma alumina has the same spinel structure as the blocks in beta alumina (ABC cubic oxygen packing), whereas alpha alumina has a corundum structure (AB hexagonal oxygen packing) [1,5,6,36].

**Figure 2:**
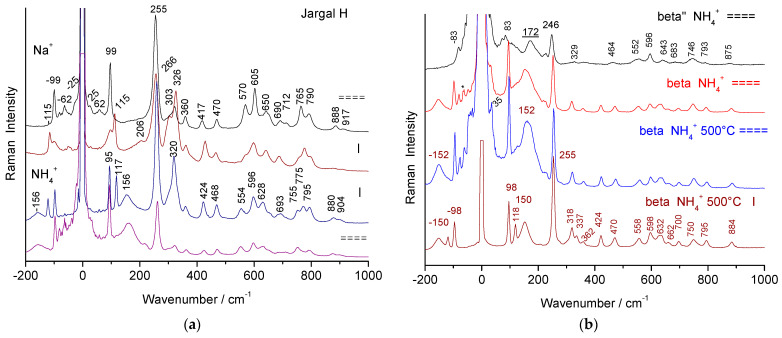
Raman spectra recorded with the 514.5 nm line using a ×50 LWD microscope objective for sodium and ammonium beta alumina single crystals from a Jargal^®^ H brick (**a**) and from laboratory synthesis (HF self-crucible technique) (**b**); spectra have been recorded with the electric vector of the laser beam in the platelet plane (===) or perpendicular (I) on Na and NH_4_ beta alumina crystals; in (**b**), a comparison is shown with the spectrum of an ammonium beta″ alumina crystal and with ‘ammonium’ beta alumina crystals thermally treated at 500 °C (crystals parallel and perpendicular to the electric vector), actually hydroxonium beta alumina. The broad mode labeled with an underlined wavenumber is mainly a fluorescence signal (* Poorly filtered stray laser lines).

Figure 2 compares polarized Raman spectra of a non-stoichiometric beta alumina single crystal in two orientations (backscattering on the platelet surface, i.e., the (a,b) plane, and on the side, parallel to the c-axis). Raman peaks above ~300–350 cm^−1^ arise from spinel block modes [3,21,23,34,35], while lower-frequency modes are attributed to Al-O-Al bridges (with stronger polarized intensity) and mobile ions. The main modes of mobile sodium and ammonium ions appear at 62 and 156 cm^−1^, with additional features below 30 cm^−1^ [21,23,32,33]. The beta″ spectrum is broadly similar, though its peaks are broader, especially those related to mobile ions [3,21,23,34].

The transformation of non-stoichiometric ammonium beta alumina into its stoichiometric form (beta-NH_4_ alumina heated at 500 °C) results in band narrowing (Figure 2), corresponding to the removal of structural disorder caused by excess oxygen atoms. These changes have been well documented [3,21,23,32].

Structural changes induced by thermal treatments at higher temperatures of stoichiometric beta alumina crystals are very sensitive to the Na/NH_4_ exchange ratio. If the Na-NH_4_ ion exchange is not complete, some stoichiometric beta alumina coexists with transition alumina and dominates the Raman spectrum due to the narrowness of its peaks and the preferential orientation of platelets. By contrast, spectra of fully exchanged crystals heated at 500 °C are free of significant residual contributions from the non-stoichiometric beta alumina structure. When the Na^+^-NH_4_^+^ ion exchange is complete, the decomposition of NH_4_^+^ ions into protons and volatile NH_3_ molecules induces a complete reaction between protons and excess oxygen ions in the conducting plane, producing water molecules. The resulting crystal becomes stoichiometric, and solvation of protons with atmospheric water during cooling gives rise to hydroxonium beta alumina [21,23,33]. Some differences are observed as a function of the crystal orientation with respect to the light electric vector, due to crystalline anisotropy. Major modifications concern modes involving Al-O-Al bridges, i.e., those at 100 and 250 cm^−1^, especially for the E_2g_ polarization (see the narrowing of the 99 cm^−1^ mode in Figure 2).

A combination of transmission electron diffraction [4,23], X-ray diffraction [21,23], and EPR [37] studies shows that gamma alumina has a unit cell six times that of beta (beta″) alumina in the (a,b) plane. It is likely that protons remain present in the structure and stabilize the transition alumina phases. A more recent study [38] identified the gamma alumina space group as Fd$\bar{3}$m with the lacunar formula Al_21+x_□_+2x_O_32_ using small crystals obtained as a product of the corrosion reaction between β-sialon and steel. 

### 3.2. Raman Spectra Evolution with the Transition from Stoichiometric Beta Alumina to Transition Alumina

**Figure 3:**
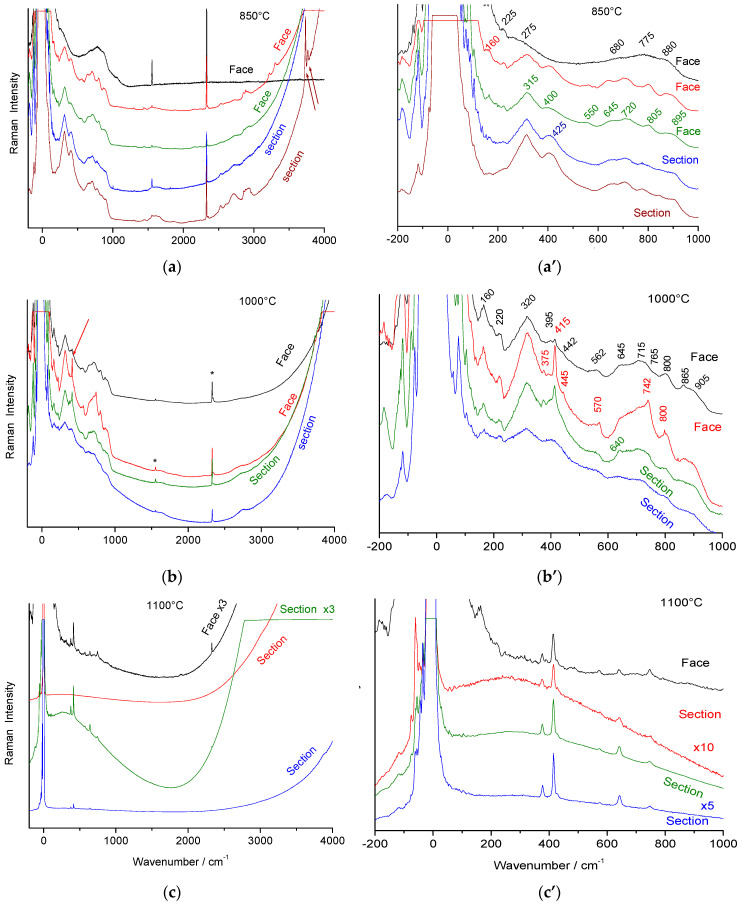
Raman spectra recorded with the 514.5 nm line using a ×50 LWD microscope objective for beta alumina single crystals (face: platelet surface; section: platelet side) heat-treated for 4 h at various temperatures. The upper series of spectra (**a**,**a’**) was recorded on ~90% ammonium-exchanged crystals; the others (**b**,**b’**,**c**,**c’**) are fully exchanged. On the right, a zoom is shown for the range of vibrational modes (* and arrow: cosmic ray).

**Figure 4:**
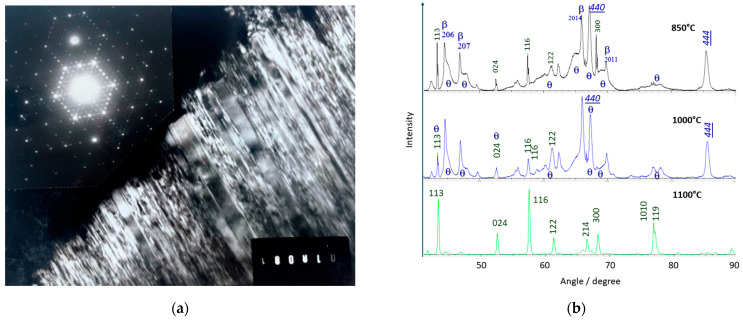
Electron diffraction (**a**) of the (**a**,**b**) plane of a thermally treated crystal at 800 °C showing the 3a and 6a superstructure, and corresponding dark-field image showing the coexistence of ordered layers with many stacking faults along the c-axis; (**b**) comparison of the X-ray diffraction patterns of (strongly powdered) stoichiometric beta alumina single crystals heated at 850, 1000, and 1100 °C (crystals powdered after thermal treatment). The main Bragg peaks are labeled for alpha (bottom spectrum), theta (broad peaks), and gamma alumina (hkl underlined), see text.

Figure 3 shows Raman signatures of ‘ammonium’ beta alumina single crystals heated at 850, 1000, and 1100 °C in air. X-ray diffraction patterns are given in Figure 4.

Previous studies [4,21,23,37] demonstrated the formation of a gamma alumina phase, ordered at long range in the (a,b) plane but with many stacking faults along the c-axis (Figure 4a). The Raman spectrum broadens and the number of bands is reduced according to the higher symmetry (hexagonal for the beta phase, cubic for the gamma phase) and the lower Z number expected for an evolution from an aluminate to a spinel structure with a simpler formula. New (broad) bands are observed. In some spots, an even broader signature with fewer modes (i.e., higher symmetry) is recorded, with peaks only at 225, 275, 690, 775, and 880 cm^−1^. The latter spectrum is quite similar to that recorded on thermally treated alumina monoliths in the 1990s with the first generation of Raman microscopes (the Mole) [2], except that at that time, the spectral range below 200 cm^−1^ was not resolved, and an additional Al(OH)_3_ signature was also detected (peak at ~360 cm^−1^). The main bands were observed at 260, 525–575, 705, 780, and 825 cm^−1^. Thus, two different transition aluminas are observed in thermally treated ammonium beta alumina single crystals. A modification of the fluorescence background is also obvious.

Figure 4b compares X-ray diffraction patterns recorded on thermally treated ammonium beta alumina crystals. The crystals were powdered, but due to their platelet shape, preferential orientation enhances some hkl peaks. After thermal treatment at 1100 °C, transformation into alpha alumina is almost complete according to the Raman data (Figure 3c’). The peaks are quite narrow, indicating good crystallinity. Surprisingly, peaks corresponding to traces of alpha alumina in materials heated at 850 °C and 1000 °C are narrower, forming a doublet (resolving a_1_ and a_2_ contributions of the X-ray source?).

In addition to traces of beta alumina, two types of alumina are observed: a well-crystallized gamma alumina phase (PDF 04-007-2283 [39], Supplementary Materials) with intense and narrow peaks and another transition alumina characterized by broad bands whose positions match those of the theta phase (PDF 04-002-2602, [39]), Supplementary Materials.

Narrow 20l peaks of pristine beta alumina are also observed. Their persistence is not surprising due to the platelet shape of the crystals. But these peaks are asymmetric and very close to those of the theta phase. Syntaxy between the different phases is therefore expected. Similar peak asymmetry, characteristic of stacking disorder, is observed in many layered structures such as kaolinite, illite, and polytypes (SiC, CdS). Electron diffraction and dark-field images (from ref. [23]) show the long-range order in the (a,b) plane of pristine beta alumina and the large disorder along the c-axis. The disorder of gamma alumina is also anisotropic because the 220, 222, and 440 peaks of the cubic phase are narrow, while the 111 and 311 peaks are broader.

As a primary conclusion, at least four phases are identified, beta, gamma, theta, and alpha alumina, with syntaxy between the first ones, consistent with the preservation of the optical clarity of the crystal before significant nucleation of alpha alumina.

The vibrational Raman spectrum of the alpha alumina phase is detectable by its main peak at ca. 417 cm^−1^, which appears at very low intensity after thermal annealing above 1000 °C. Simultaneously, the crystal loses its optical clarity and nucleation becomes visible under the microscope (Figure 1b).

**Figure 5:**
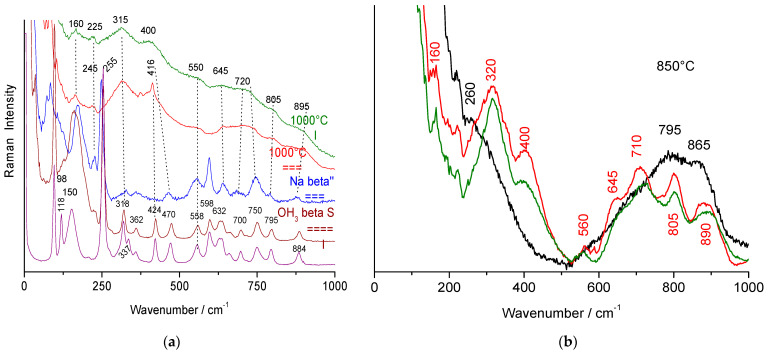
Comparison of the Raman spectra of transition and beta alumina: (**a**) dotted lines are guides for the eye; (**b**), spectra after baseline subtraction: in black, theta alumina; in red and green, gamma alumina.

Comparison of the Raman spectra in Figure 5a shows that peak wavenumbers of spinel block modes are almost unchanged. The main difference between beta alumina and gamma alumina spectra is the disappearance of the strong Al-O-Al bridge modes (~100 and 255 cm^−1^). These strong modes at relatively low energy are characteristic of a layered structure and obviously disappear in a packed structure. We can assume that the spectra with a limited number of very broad Raman modes correspond to the phase with very broad Bragg peaks, and the spectra with a higher number of bands correspond to gamma alumina crystals with rather narrow 440 and 444 Bragg peaks (Figure 4b).

Subtraction of the baseline (using the simplest solution, two line segments, Figure 5b) allows a better comparison of the two types of spectra with spinels or related structures. The differentiation between the two types of transition alumina is more obvious than in the raw spectra.

The spectra of spinel phases of transition metal oxides (Fe, Ni, Co, Cr), whose M-O bond is more covalent than the Al-O bond, exhibit Raman spectra characterized by an intense stretching mode of the XO_4_ tetrahedra between 650 and 700 cm^−1^ [40]. If several types of tetrahedra exist, the mode broadens and several components appear [40]. The wavenumber of the M-O stretching mode is a slightly higher because aluminum is lighter. At lower energy, bending modes strongly coupled to the external R’ and T’ modes (tetrahedra sharing vertices and edges) are observed. Thus, we observe the signature of the two different transition aluminas, evidenced either by XRD and Raman techniques, for instance, the gamma and theta phases.

The spectrum that exhibits fewer components in the spectral range expected for stretching modes is assigned to theta alumina. The other spectrum, with many modes between 500 and 1000 cm^−1^, very similar to those of beta alumina, is assigned to gamma alumina (common spinel structure). Raman spectroscopy is very sensitive to orientational disorder of XO_4_ tetrahedra and the presence of vacancies. It is therefore not surprising that the lacunar gamma phase, which exhibits relatively narrow Bragg peaks for some hkl reflections (better long-range translational order in the planes derived from (a,b) beta alumina), has a broader spectrum than stoichiometric beta alumina but with narrower Raman bands than the theta phase. The large Rayleigh wing indicates strong dielectric heterogeneity.

### 3.3. Comparison with Raman Spectra of Alumina Xerogel and Glass

**Figure 6:**
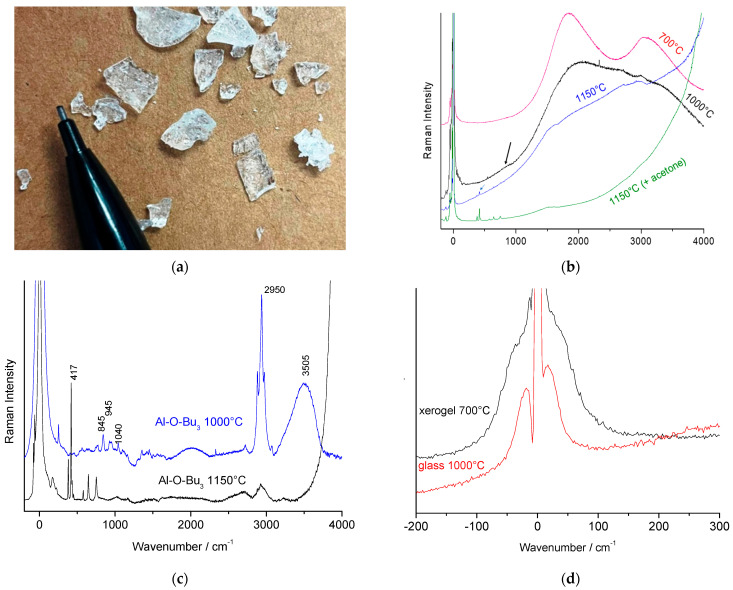
(**a**) Optically clear alumina glass pieces annealed at 700 °C, prepared by slow hydrolysis of Al-butoxide; representative full spectral range (**b**) and (**c**) baseline-subtracted Raman spectra (c, ×50 objective) recorded on optically clear alumina glass annealed at 1000 °C and 1150 °C. A zoom of the low-wavenumber anti-Stokes and Stokes range (**d**) compares an alumina glass heated at 700 °C and 1000 °C (as-recorded spectra, ×50 objective).

Figure 6 shows the spectra of thermally treated, optically clear amorphous ‘alumina’ monoliths (Figure 6b) prepared by slow hydrolysis of aluminum alkoxide [2,22].

The vibrational spectrum of the alpha alumina phase (peaks at 380m, 417S, 430w, 450vw, 576w, 750m, 845S, and 895w cm^−1^; m: medium; S: strong; v: very; w: weak [1]) can be identified only by its stronger band at 417 cm^−1^, which appears at very low intensity after thermal annealing above 1000 °C. (Figure 6a, blue small arrow). For samples treated at lower temperatures, the dominant features arise from fluorescence (as evidenced by the lack of a symmetrical counterpart on the anti-Stokes side and by changes when using a different laser line).

The gel heat-treated at 1000 °C only shows a tiny broad band around 800 cm^−1^ (black arrow in Figure 6). It is not possible to distinguish between gamma and theta phases at this stage.

Subtracting the background caused by fluorescence—a point discussed later—reveals additional spectral features with varying intensities. However, background subtraction is inherently subjective, as it depends on the chosen procedure (here, line-segment fitting was used for simplicity). This approach alters the spectrum by removing very broad contributions.

Since the materials were prepared by liquid chemical routes using organic reagents, weak modes characteristic of aliphatic chains ((C-(H)_n_ stretching modes around 2850–2950 cm^−1^) and aromatic rings (around 3060 cm^−1^ [2,22,41]) are observed for thermal treatments below 1150 °C (Figure 6c). A broad band with maximum intensity between 3500 and 3600 cm^−1^ is also observed, corresponding to stretching modes of protonic species (water molecules, oxonium or hydroxyl ions) adsorbed on the oxide surface [42,43,44,45]. Typically, water molecule modes appear between 3200 and 3400 cm^−1^, oxonium ion modes around 3400–3500 cm^−1^, and O-H^-^ ion modes above 3600–3700 cm^−1^ [42,43,44]. The bandwidth of protonic species bands is expected to decrease as the center of gravity shifts to higher wavenumbers [42]. The study of homologous signatures obtained by deuteration, and in particular by H/D isotope dilution, is necessary for more precise attribution [45].

The intensity of these bands depends on the accessible surface area to protonated species, i.e., microporosity or vacancies, and is thus inversely proportional to the degree of densification. Organic residues disappear between 800 and 900 °C depending on the heat-treatment conditions, in air, while protonic species persist until higher temperatures, between 900 and 1000 °C. This departure temperature is consistent with similar oxide materials [2,43,44,45,46,47]. Typically, the final densification of oxides obtained by wet chemical routes is driven by the removal of residual protons [47].

After baseline subtraction, wide or weakly structured bands of very low intensity become visible between ~500 and 1200 cm^−1^. This range corresponds to vibrational contributions expected for Al-O modes in a spinel-like structure, as observed in thermally treated beta alumina [3,4,21]. Additionally, a broad weak band around 180 cm^−1^, if the Rayleigh wing is sufficiently weak, appears to be related to the fluorescence background. However, vibrational bands remain very faint. Spectra recorded on heated single crystals are clearly much stronger and more reliable.

We observe the progressive disappearance of organic residues and protonated species, along with an increase in the continuous background, as the heat-treatment temperature increases. This continuous background is intrinsic, not caused (as often assumed) by fluorescence from organic residues on microporous surfaces. No traces of AlO(OH), characterized by its main peak at 363 cm^−1^ [48], were detected.

Figure 6d details the low-wavenumber domain closest to the elastic Rayleigh peak. As expected for alumina xerogels (composition Al_2_O_2.5_(OH)_0.9_, 0.4H_2_O according to [46]), Rayleigh wings are observed on both the Stokes and anti-Stokes sides. A gel, being physically a frozen liquid, exhibits a Raman signature similar to that of a liquid [48,49,50,51].

For higher-temperatures treatments (800–1000 °C), a Boson peak at 20–50 cm^−1^ is observed, characteristic of the glassy state (composition A1_2_O_2.8_(OH)_0.35_. 0.3H_2_O according to [46]) [52,53,54,55]. The Boson peak observed at 800 °C disappears at higher temperatures, coinciding with the progressive disappearance of the Rayleigh wings. Two hypotheses may explain this: (i) modification of the glassy phase structure, causing the Boson peak to shift to lower wavenumbers and become unresolved at the instrument’s resolution); (ii) a decrease in the amount of the glassy phase as a new phase forms and grows.

Examination of the 300–700 cm^−1^ range shows increasing intensity of the alpha alumina Raman signature (Figure 4), but only for treatments above 1100 °C. An intermediate phase without a visible Raman signature is thus expected. It is well established that many disordered phases, known as transition aluminas, form at temperatures below that of alpha alumina [56,57].

The sequence of spectral changes is summarized in Table 2.

### 3.4. Fluorescence Signatures

**Figure 7:**
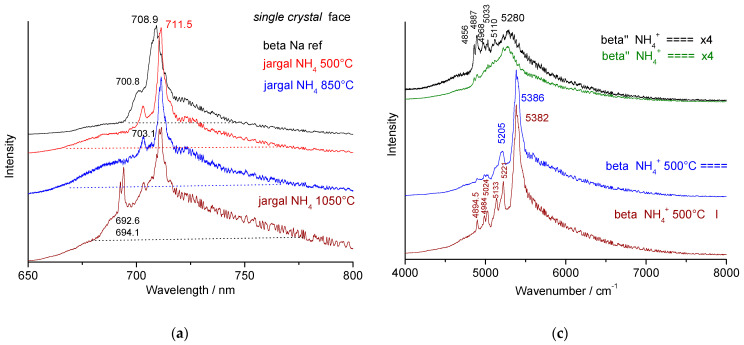

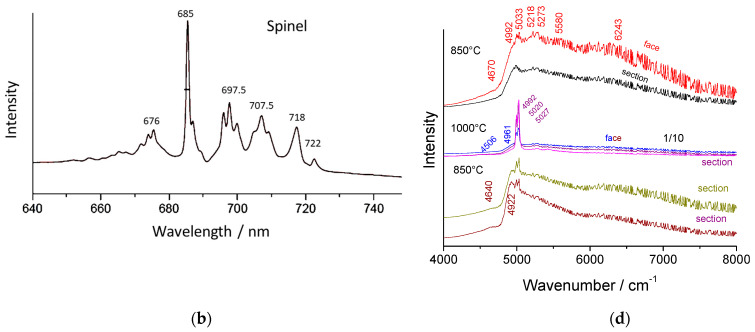
Fluorescence of chromium in crystals of (**a**) non-stoichiometric sodium beta alumina (beta Na ref, top) and stoichiometric beta ammonium alumina thermally treated at various temperature (Jargal NH_4_ 500 °C, 850 °C and 1050 °C); (**b**) spectrum of MgAl_2_O_4_ single crystal; (**c**) ammonium beta alumina single crystal (measurements on the face (===) or on the edge section (I)) heat-treated at different temperatures, and comparison with beta″ alumina; (**d**) fluorescence spectra recorded on crystals heated at 850 °C (mainly theta phase) and 1000 °C (mainly gamma phase plus nucleation of alpha phase; a spot corresponding to theta phase is given at the bottom. The spectra are presented according to the absolute scale (648 to 800 nm wavelength, left side) or according to the relative Raman scale (4000–8000 cm^−1^ for excitation at 514.5 nm, right side). See Table 3 for values on both scales.

In Figure 3 and Figure 6, the increase in the background with increasing wavenumber, together with the appearance of the alpha alumina Raman signature, is evident. We assign this phenomenon to the low-wavelength ‘wing’ of chromium fluorescence. Figure 7 compares the 4000 to 8000 cm^−1^ spectral range (Raman shift scale), corresponding to the 650–800 nm wavelength range (absolute scale). It shows a similar spectral range for sodium and incompletely exchanged non-stoichiometric beta and beta’’ alumina single crystals, as well as for the stoichiometric phases obtained by thermal treatment of pure ammonium forms. For comparison, the fluorescence spectrum of MgAl_2_O_4_ is also shown with a main peak at 685 nm and narrow associated peaks.

**Figure 8:**
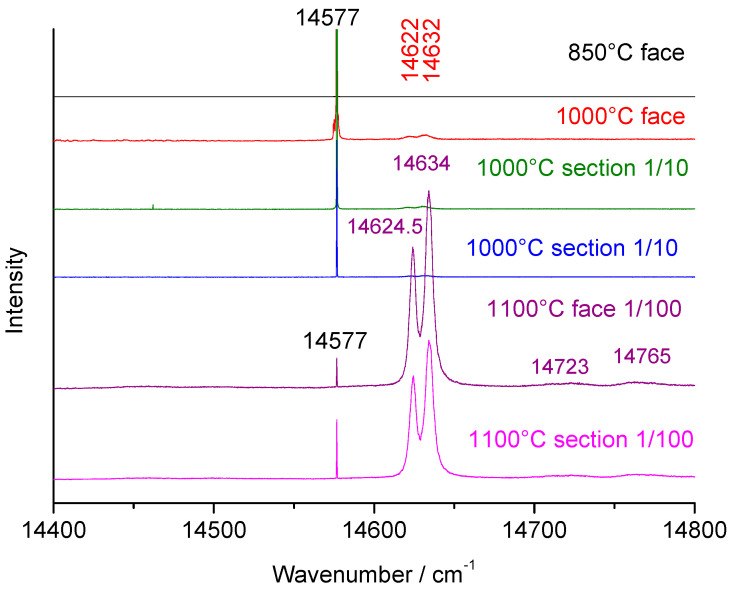
Fluorescence of chromium in rystals of stoichiometric beta ammonium alumina (Jargal H^®^) single crystal (measurements on the face (===) or on the section (I)) heat-treated at different temperatures according to two polarizations (face (parallel) and section (perpendicular)). D1 (1/10) and D2 (1/100) optical density filters are used when the signal is very strong.

Three types of features are observed:

(i) The well-known very narrow doublet of Cr-fluorescence in corundum at 5002 and 5037 cm^−1^ (Raman shift scale) or 692.6 and 694.1 nm (absolute scale) [5,6,15,16,17,18,58,59,60,61] (another doublet at ~14,600 cm^−1^ (2068 nm), shown in Figure 8, will be discussed later);
(ii) A broader peak around 709 nm (5380 cm^−1^), characteristic of non-stoichiometric (NS) beta alumina, which shifts to 711.5 nm (5386 cm^−1^) for stoichiometric (S) beta alumina;
(iii) Very broad bands with maxima at 706.3 nm (5280 cm^−1^) for ammonium beta″ alumina, 712.5 nm (5400 cm^−1^) for thermally treated ammonium beta alumina, and 692.7–694 nm (5000–5027 cm^−1^) for alumina thermally treated at 1150 °C and above.

Each phase can be identified by its fluorescence signature; all peak positions are listed in Table 3. The non-stoichiometric beta″ form is more disordered [23,24], resulting in a broader signal than the non-stoichiometric beta phase (Figure 7c). A small shift of the main peak is observed. The slight shift and narrowing of the main peak from non-stoichiometric Na beta to stoichiometric OH_3_ beta alumina (i.e., 500 °C heat-treated NH_4_ single crystal, Figure 7) arise from the disappearance of disorder in the spinel block. In both cases, a broad band below the narrow peak is also observed. The small, relatively narrow peak is expected to be associated with the stronger one.

Thermal treatment above 700 °C increases the intensity of the broader component, corresponding to the formation of disordered transition alumina(s). In the Jargal H^®^ 1050 °C heat-treated sample (Figure 7a), the narrow peaks of Na and NH_4_ non-stoichiometric phases remain due to incomplete crystal exchange.

In addition to the main peaks, a broad base and narrow, lower-intensity peaks are observed. These latter peaks likely arise from structural defects involving chromium atoms in the spinel blocks or from very minor phases (excluding alpha phase, see Table 3). The broad components are probably associated with disorder in the conductive planes, an intrinsic characteristic of beta and beta″ structures, which are fast ion conductors.

Figure 7d shows the most representative fluorescence spectra associated with the representative Raman spectra of transition alumina shown in Figure 5b. Two types of very broad spectra are observed: narrow (4992–5273 cm^−1^, 692.3–706 nm) and broad (4640–7500 cm^−1^, 675.8–837.8 nm). For samples annealed at 1000 °C and above, the characteristic doublet of alpha alumina (5020–5027 cm^−1^, i.e., 693.7–694 nm), along with a pre-peak at 4992 cm^−1^ (692.3 nm), appears and dominates with further heating, becoming practically the only feature above 1150 °C. The spectra vary little depending on polarization (measurement on the face or edge of the wafers), but the intensities of the components vary. As with vibrational modes, two types of signatures are observed for the different forms of transition alumina, gamma and theta, consistent with previous studies [6,8]. Differences between the spectra of the two transition alumina phases are, however, weak.

The fluorescence spectra in the 4000–8000 cm^−1^ (~650–875 nm, Figure 7) range are preferred here compared to those around 14,600 cm^−1^ (Figure 8). The doublet at 14,624.5–14,634 cm^−1^, (2078.2–2082.3 nm) is also characteristic of alpha alumina [15,16,17,18]. Components at lower energy are more intense and easier to record with standard spectrometers. An extremely narrow peak is observed at 14,577 cm^−1^ (2057.9 nm), which decreases as the alpha alumina signature increases (except in one crystal heated at 1000 °C); its bandwidth is comparable to other fluorescence peaks, and its position is slightly shifted, possibly corresponding to another phase. Wen et al. [6] assigned the 14,575 and 14,645 cm^−1^ doublet to theta transition alumina. Their spectra differ somewhat from ours. In our case, the 14,624.5–14,634 cm^−1^ doublet corresponds to alpha alumina.

**Figure 9:**
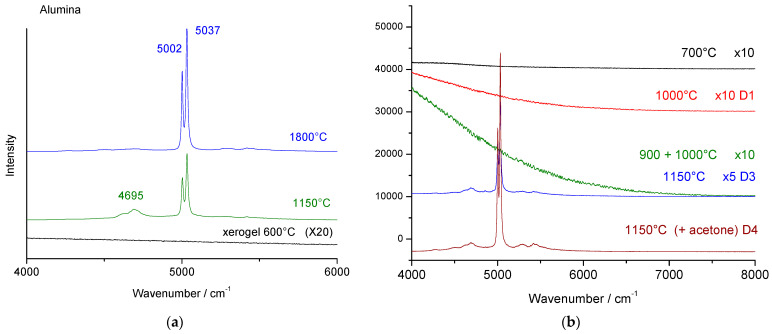
Fluorescence of chromium in optically clear alumina gel heated at 1150, and 1800 °C ((**a**), relative wavenumber scale) and (**b**) 700, 1000, 900 + 1000, and 1150 °C. Due to the huge variation of the spectrum intensity, they have been recorded using D1 (1/10), D3 (1/1000), and D4 (1/10,000) filters and multiplied by 5 or 10 in order to allow visualization.

No significant fluorescence is observed for amorphous alumina xerogel and glass in the 4000–8000 cm^−1^ range (Figure 9). Instead, fluorescence occurs at lower energies, within the 1000–4000 cm^−1^ range. As shown in Figure 6a, the xerogel exhibits two bands at ~2000 and ~3000 cm^−1^ (573.5–608.5 nm), of which only the first persists in the glassy phase after thermal treatment between 800 and 1000 °C. Fluorescence associated with chromium in the spinel local structure appears only for materials heated above 1100 °C, i.e., upon nucleation of the alpha alumina phase. Compared with ammonium beta alumina, the temperature window—if it exists—where transition aluminas develop is narrower in thermally treated alumina monoliths obtained by hydrolysis-polycondensation of aluminum alkoxides.

Notably, a specific component at 4695 cm^−1^ (678.3 nm) is observed for monoliths heated at 1150 °C. This feature, appearing just below the band observed for the crystal, may correspond to the formation of another transition alumina. It can thus be assumed that, at this temperature, a phase distinct from the γ-alumina form develops.

The remarkable intensity of the chromium fluorescence signal in alpha alumina, which can vary by several orders of magnitude between the nucleation of the alpha phase and its complete crystallization, makes it an effective probe for detecting and quantifying alpha alumina formation. This is useful, for example, in the thermal treatment of alumina fiber precursors (to be reported in a companion paper [62]) and in porcelain during the transformation of mullite 2Al_2_O_3_·SiO_2_ (2:1) into mullite 3Al_2_O_3_·2SiO_2_ (3:2), providing a measure of the firing degree [63]. The technique also enables operando measurements, with less than a second being sufficient to obtain very good spectra.

Although the spectra of disordered phases, such as transition alumina and amorphous alumina, are much broader, the chromium fluorescence signal remains useful for characterizing these phases and monitoring their evolution—for example, during the application of stress [64]. Although numerous studies have focused on the use of transition aluminas as catalyst supports [65,66,67,68,69,70,71], including spectroscopic investigations by Raman technique [70], the limited knowledge of their optical spectra remains a potential source of error. The present work seeks to contribute to a clearer understanding of these spectra

## 4. Conclusions

Alumina and aluminate phases can be identified from their chromium fluorescence spectra. The fluorescence signal, extremely intense and sharp for the doublets characteristic of the alpha alumina phase (around 5000 and 14,600 cm^−1^), is particularly effective for detecting the formation of this phase. The different beta or beta″ alumina phases, whether of varying stoichiometry or composition (e.g., sodium aluminate, ammonium aluminate), also exhibit specific signatures that are more complex than that of alpha alumina. Certain peaks are likely associated with structural defects, and a precise combined study of each crystal using diffraction, fluorescence, and EPR spectroscopy would be required for their identification. Transition aluminas exhibit broader spectra with several components. In this work, we identify the spectra of the gamma and theta transition aluminas, with results consistent with the preliminary data of Renush et al. [6] for the theta phase. Another transition phase is inferred for thermally treated alumina glass.

High-quality vibrational Raman spectra of the θ and γ phases were also obtained here for the first time, confirming the presence of protonic species in transition aluminas. It is likely that the formula of transition alumina—or more precisely, of proton aluminate—is more complex than previously proposed, requiring explicit mention of protons: Al_2+y/3-x/3_□_2y_ H_x_O_3+z_. Reliable spectra, however, are only obtained for transition alumina crystals derived from thermally treated ammonium β-alumina, and the Raman spectra reported here represent the first high-quality spectra available for these phases. They can therefore be considered as reference data.

Finally, the observation of a Boson peak can be taken as evidence for the possibility of preparing glassy alumina (in this case also containing some protons).

## Figures and Tables

**Table 1:** Studied samples (diameter of 1 Euro cent: 16.25 mm).

Samples	View	Synthesis	Thermal Treatments	Remarks (Composition)	Refs.
Na beta alumina single crystal	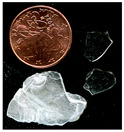	Cooling from the melt	no	Non-stoichiometric (11 Al_2_O_3_ 1.3 Na_2_O)	[22,23]
Na beta″ alumina single crystal	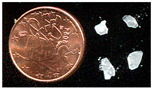	Cooling from the melt	no	Non-stoichiometric (11 Al_2_O_3_ 1.6 Na_2_O)	[22,23]
NH_4_ beta alumina single crystal	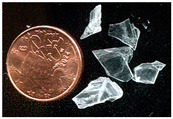	Ion exchange	~180 °C	Non-stoichiometric (11 Al_2_O_3_ 1.3 (NH_4_)_2_O)	[22,23]
NH_4_ beta″ alumina single crystal	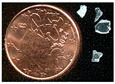	Ion exchange	~180 °C	Non-stoichiometric (11 Al_2_O_3_ 1.3 (NH_4_)_2_O)	[22,23]
NH_4_ beta alumina single crystal	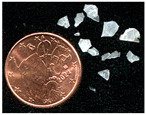	Ion exchange + thermal treatment	500 °C	Stoichiometric (11 Al_2_O_3_ (OH_3_)_2_O)	[22,23]
OH_3_ beta alumina crystal	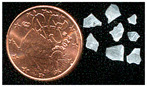	Ion exchange + thermal treatment	750 °C	Phase transformation (Al_2_O_3_ (H_n_))	[3,21,23]
H^+^ transition alumina	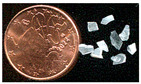	Ion exchange + thermal treatment	850 °C	Transition Alumina (Al_2_O_3_ (H_n_))	
Alpha/transition alumina	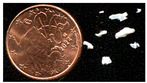	Ion exchange + thermal treatment	1000 °C	(Al_2_O_3_)	
Alumina xerogel	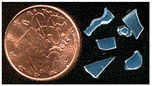	Very slow hydrolysis-polycondensation + thermal treatment	700 °C	Optically clear monolith (Al_2_O_2.5_(OH)_0.9_, 0.4H_2_O)	[2,23]
Alumina glass	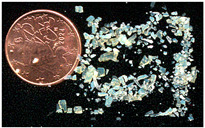	Very slow hydrolysis-polycondensation + thermal treatment	1000 °C	Phase transformation (A1_2_O_2.8_(OH)_0.35_, 0.3H_2_O)	[2,23]
Alumina (nanocrystalline)	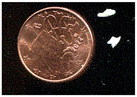	Very slow hydrolysis-polycondensation + thermal treatment	1100 °C	A1_2_O_3_	[2,23]

**Table 2:** Phase transformations evidenced from Raman and Rayleigh spectra.

Phase (Characteristics)	T	Phase (Characteristics)	T	Phase (Characteristics)	T	Phase (Characteristic)	T	Phase (Characteristic)
Chemically prepared ‘gel’ (Rayleigh wing; CH_n_ modes, O-H modes)	<800 °C	Microporous protonated glassy alumina (Boson peak; strong O-H modes)	800 °C –900 °C	Deprotonated transition alumina (Rayleigh wing; weak O-H modes)	900–1000 °C	Transition alumina Nucleation of alpha alumina (alpha alumina peaks; disappearance of Rayleigh wing)	1050 °C –1100 °C	Growth of alpha alumina at the expand of transition alumina (alpha alumina peaks, fluorescence background)
S beta alumina single crystal	850 °C	Syntaxic formation of gamma and theta alumina single crystals			1000 °C	Development of gamma alumina and nucleation of alpha alumina	1100 °C	

**Table 3:** Peak position of fluorescence peaks (in *italics* after Renusch et al. [8], in **bold** residual beta and beta’’ alumina phases).

Beta Na		Beta’’ NH_4_ 500 °C		Beta-1 NH_4_ 500 °C		Beta-2 NH_4_ 500 °C		J NH_4_ 500 °C		Beta NH_4_ 850 °C		J NH_4_ 1050°		Beta NH4 1000		A 1050 °C				
cm^−1^	nm	cm^−1^	nm	cm^−1^	nm	cm^−1^	nm	cm^−1^	nm	cm^−1^	nm	cm^−1^	nm	cm^−1^	nm	cm^−1^	nm	cm^−1^	nm	
																		4294	660.4	
														4506				4499.8	669.5	
																		4570.9	672.7	
																		4643.5	676	
										4670						4665	677			
																4695	678.4			
		*4856*	*685.8*																	Θ
		*4887*	*687.3*			*4894.5*	*687.7*							4922						
														4961						
		4968	691.2																	
						4984	691.9			4992			**692.6**	4992		**5002**	**692.8**	**5002**	**692.8**	**β**
														**5020**						
		5033	694.3			5024	693.8			5033			**694.1**	**5027**		**5037**	**694.5**	**5035.5**	**694.4**	**β**
		5110	698																	
						5133	699.1													
				5205	702.7															
						5221	703.5	52,135	703.1	5218		5213.5	703.1					5211.6	703	
5166.9	700.8									5273										**β NS**
**5330**	**708.9**																			**β NS**
		**5280**	**706.4**																	**β″ NS**
				**5382**	**711.5**	**5386**	**711.7**	5381.5	711.5	53,815	711.5	5381.5	711.5							**β S**
																		5282.1	706.5	
																		5411.1	713	
																		5460.1	715.5	
5545.5	720	5606	5545.5	5605	5545.5	5605		5545.5	720	5545.5	720									
										5580										
										6243										

## Data Availability

All data are in the figures.

## Supplementary Materials

The following supporting information can be downloaded at: https://www.mdpi.com/article/10.3390/ma18204682/s1, Figure S1: Indexed XRD pattern of ammonium beta alumina powdered crystals thermally treated at 850 °C, 1000 °C and 1100 °C. All other data are included in the paper.

## References

[1] Porto S.P.S., Krishnan R.S. (1967). Raman effect of corundum. J. Chem. Phys..

[2] Colomban P. (1989). Structure of oxide gels and glasses by infrared and Raman scattering: Part 1 Alumina. J. Mater. Sci..

[3] Colomban P., Lucazeau G. (1980). Vibrational study of and conduction mechanism in β alumina. I. Stoichiometric β alumina. J. Chem. Phys..

[4] Colomban P.H. (1988). Raman study of the formation of transition alumina single crystal from protonic β/β″ aluminas. J. Mater. Sci. Lett..

[5] Remush D., Grimsditch M., Jorgensen J.D., Hodges J.P. (2001). Pressure Dependence of Cr^3+^ Fluorescence in θ-Alumina. Oxid. Met..

[6] Wen Q., Lipkin D.M., Clarke D.R. (1998). Luminescence Characterization of Chromium-Containing theta-Alumina. J. Am. Ceram. Soc..

[7] Clarke D.R., Christensen R.J., Tolpygo V. (1997). The evolution of oxidation stresses in zirconia thermal barrier coated superalloy leading to spalling failure. Surf. Coat. Techn..

[8] Singh J.P., Nair B.G., Renusch D.P., Sutaria M.P., Grimsditch M.H. (2001). Damage evolution and stress analysis in zirconia thermal barrier coatings during cyclic and isothermal oxidation. J. Am. Ceram. Soc..

[9] Bunsell A.R., Berger M.H. (2000). Fine diameter ceramic fibres. J. Eur. Ceram. Soc..

[10] Bunsell A.R. (1988). Fibre Reinforcements for Composite Materials.

[11] Schneibel J.H., George E.P., McKamey C.G., Ohriner E.K., Santella M.L., Carmichael C.A. (1991). Fabrication and tensile properties of continuous-fiber reinforced Ni_3_Al–Al_2_O_3_ composites. J. Mater. Res..

[12] Lavaste V., Berger M.H., Bunsell A.R., Besson J. (1995). Microstructure and mechanical characteristics of alpha-alumina-based fibres. J. Mater. Sci..

[13] Gouadec G., Karin S., Wu J., Parlier M., Colomban P. (2001). Physical Chemistry and mechanical imaging of ceramic-fibre-reinforced ceramic-or metal-matrix composites. Compos. Sci. Technol..

[14] Redonnet J. (2025). Relation Entre Microstructure et Propriétés Mécaniques D’une Fibre D’alumine Continue en Développement. Ph.D. Thesis.

[15] Pezzotti G., Sbaizero O., Sergo V., Muraki N., Maruyama K., Nishida T. (1998). In situ measurements of frictional bridging stresses in alumina using fluorescence spectroscopy. J. Am. Ceram. Soc..

[16] Pezzotti G. (1999). In situ study of fracture mechanisms in advanced ceramics using fluorescence and Raman microprobe spectroscopy. J. Raman Spectrosc..

[17] Sinclair R., Young R.J., Martin R.D.S. (2004). Determination of the axial and radial fibre stress distributions for the Broutman test. Compos. Sci. Technol..

[18] Gouadec G., Colomban P., Piquet N., Trichet M.F., Mazerolles L. (2005). Raman/Cr^3+^ fluorescence mapping of a melt-grown Al_2_O_3_/GdAlO_3_ eutectic. J. Eur. Ceram. Soc..

[19] Jayaraman A. (1983). Diamond anvil cell and high-pressure physical investigations. Rev. Mod. Phys..

[20] Vos W.L., Schouten J.A. (1991). On the temperature correction to the ruby pressure scale. J. Appl. Phys..

[21] Colomban P., Boilot J.-P., Kahn A., Lucazeau G. (1978). Structural Investigation of Protonic Conductor NH_4_^+^ Beta Alumina and Stoichiometric H_3_O^+^ beta Alumina. Nouv. J. Chim..

[22] Colomban P. (1989). Structure of oxide gels and glasses by infrared and Raman scattering: Part 2 Mullites. J. Mater. Sci..

[23] Colomban P. (1979). Contribution à L’étude des Mécanismes de Conductivité dans les Composes de Type β et β’’ Al_2_O_3_. Ph.D. Thesis.

[24] Collin G., Boilot J.P., Colomban P., Comes R. (1986). Host lattices and superionic properties in β-and β’’-alumina. I. Structures and local correlations. Phys. Rev. B.

[25] Bragg W.L., Gottfried C., West J. (1931). The structure of β alumina. Zeitschr. Krist.-Cryst. Mater..

[26] Hao C.H., Chase L.L., Mahan G.D. (1976). Raman scattering in β−alumina. Phys. Rev. B.

[27] Frech R., Bates J.B. (1979). Raman, ir reflection, and emission spectra of sodium β-alumina. Spectrochim. Acta Part A Mol. Spectrosc..

[28] Bates J.B. (1980). Raman scattering from NH_4_^+^ and ND_4_^+^ in beta-alumina. J. Chem. Phys..

[29] Hayes W., Holden L., Tofield B.C. (1980). Infrared studies of hydrogen beta alumina. J. Phys. C Solid State Phys..

[30] Colomban P., Fillaux F., Tomkinson J., Kearley G.J. (1995). Inelastic neutron-scattering study of proton dynamics in β-alumina. Solid State Ion..

[31] McWhan D.B., Shapiro S.M., Remeika J.P., Shirane G. (1975). Neutron-scattering studies on beta-alumina. J. Phys. C Solid State Phys..

[32] Colomban P. (1981). Vibrational study of hydrogen beta alumina. J. Phys. C Solid State Phys..

[33] Lassègues J.C., Fouassier M., Baffier N., Colomban P., Dianoux A.J. (1980). Neutron scattering study of the proton dynamics in NH+ 4 and OH^+^ 3 β alumina. J. Phys..

[34] Lucazeau G. (1983). Infrared, Raman and neutron scattering studies of β-and β″-alumina: A static and dynamical structure analysis. Solid State Ion..

[35] Dohy D., Lucazeau G., Bougeard D. (1983). Vibrational and normal-mode analysis of stoichiometric β-Al_2_O_3_. Solid State Ion..

[36] Łodziana Z., Parliński K. (2003). Dynamical stability of the α and θ phases of alumina. Phys. Rev. B.

[37] Colomban P., Vivien D. (1983). EPR study of ordering in Stoichiometric β-aluminate. Phys. Stat. Sol. (a).

[38] Smrčok Ľ., Vratislav L., Křesťan J. (2006). γ-Alumina: A single-crystal X-ray diffraction study. Cryst. Struct. Commun..

[39] International Centre for Diffraction Data. https://www.icdd.com.

[40] Cvejic Z., Rakic S., Kremenovic A., Antic B., Jovalekic C., Colomban P. (2006). Nanosize ferrites obtained by ball milling: Crystal structure, cation distribution, size-strain analysis and Raman investigations. Solid State Sci..

[41] Koenig J.L. (1971). Raman scattering of synthetic polymers—A review. Appl. Spectrosc. Rev..

[42] Colomban P. (1992). Proton Conductors: Solids, Membranes and Gels-Materials and Devices.

[43] Colomban P. (2013). Proton and protonic species: The hidden face of solid-state chemistry. How to measure H-content in materials?. Fuel Cells.

[44] Tournié A., Ricciardi P., Colomban P. (2008). Glass corrosion mechanisms: A multiscale analysis. Solid State Ion..

[45] Colomban P. (2023). Vibrational characterization of the various forms of (solvated or unsolvated) mobile proton in the solid state. Advantages, limitations and open questions. Solid State Ion..

[46] Vendange V., Colomban P. (1996). Determination of the hydroxyl content in gels and porous “glasses” from alkoxide hydrolysis by combined TGA and BET analysis. J. Porous Mater..

[47] Colomban P., Vendange V. (1992). Sintering of alumina and mullites prepared by slow hydrolysis of alkoxides: The role of the protonic species and pore topology. J. Non-Crystall. Solids.

[48] Doss C.J., Zallen R. (1993). Raman studies of sol-gel alumina: Finite-size effects in nanocrystalline AlO(OH). Phys. Rev. B.

[49] Venkateswarlu K., Thyagarajan G. (1959). Intensity studies in Raman effect: Part II. On the wing accompanying the Rayleigh line in liquids and liquid mixtures. Z. Phys..

[50] Perrot M., Brooker M.H., Lascombe J. (1981). Raman light scattering studies of the depolarized Rayleigh wing of liquids and solutions. J. Chem. Phys..

[51] Gochiyaev V.Z., Malinovsky V.K., Novikov V.N., Sokolov A.P. (1991). Structure of the Rayleigh line wing in highly viscous Iiquids. Philos. Mag. B.

[52] Cortie D.L., Cyster M.J., Ablott T.A., Richardson C., Smith J.S., Iles G.N., Wang X.L., Mitchell D.R.G., Mole R.A., de Souza N.R. (2020). Boson peak in ultrathin alumina layers investigated with neutron spectroscopy. Phys. Rev. Res..

[53] Roy A., Sood A.K. (1995). Phonons and fractons in sol-gel alumina: Raman study. Pramana.

[54] Malinovsky V.K., Sokolov A.P. (1986). The nature of boson peak in Raman scattering in glasses. Solid State Commun..

[55] Schroeder J., Wu W., Apkarian J.L., Lee M., Hwa L.G., Moynihan C.T. (2004). Raman scattering and Boson peaks in glasses: Temperature and pressure effects. J. Non-Crystall. Solids.

[56] Macêdo M.I.F., Bertran C.A., Osawa C.C. (2007). Kinetics of the γ → α-alumina phase transformation by quantitative X-ray diffraction. J. Mater. Sci..

[57] Santos P.S., Santos H.S., Toledo S.P.D. (2000). Standard transition aluminas. Electron microscopy studies. Mater. Res..

[58] Krishnan R.S. (1947). Raman spectrum of alumina and the luminescence of ruby. Proc. Indian Acad. Sci.—Sect. A.

[59] Zvonarev S.V., Smirnov N.O. (2019). Luminescence quenching in magnesium-doped alumina ceramics. Phys. Solid State.

[60] Choudhari K.S., Hebbar D., Kulkarni S.D., Santhosh C., George S.D. (2019). Cr^3+^ doped nanoporous anodic alumina: Facile microwave assisted doping to realize nanoporous ruby and phase dependent photoluminescence. Ceram. Int..

[61] Jankowiak R., Roberts K., Tomasik P., Sikora M., Small G.J., Schilling C.H. (2000). Probing the crystalline environment of α-alumina via luminescence of metal ion impurities: An optical method of ceramic flaw detection. Mater. Sci. Engn. A.

[62] Redonnet J., Colomban P., Berger M.H., Joannès S., Francy O.

[63] Colomban P., Hsieh J., Shi C.-F.

[64] Frankberg E.J., Lambai A., Zhang J., Kalikka J., Khakalo S., Paladino B., Cabrioli M., Mathews N.G., Salminen T., Hokka M. (2023). Exceptional microscale plasticity in amorphous aluminum oxide at room temperature. Adv. Mater..

[65] Cocke D.L., Johnson E.D., Merrill R.P. (1984). Planar models for alumina-based catalysts. Catal. Rev. Sci. Engn..

[66] Rashkeev S.N., Sohlberg K., Glazoff M.V., Novak J., Pennycook S.J., Pantelides S.T. (2003). Transition metal atoms on different alumina phases: The role of subsurface sites on catalytic activity. Phys. Rev. B.

[67] Trueba M., Trasatti S.P. (2005). γ-Alumina as a support for catalysts: A review of fundamental aspects. Eur. J. Inorg. Chem..

[68] Wachs I.E., Roberts C.A. (2010). Monitoring surface metal oxide catalytic active sites with Raman spectroscopy. Chem. Soc. Rev..

[69] Kovarik L., Bowden M., Szanyi J. (2021). High temperature transition aluminas in δ-Al_2_O_3_/θ-Al_2_O_3_ stability range. J. Catal..

[70] Hess C. (2021). New advances in using Raman spectroscopy for the characterization of catalysts and catalytic reactions. Chem. Soc. Rev..

[71] Yang Y., Miao C., Wang R., Zhang R., Li X., Wang J., Wang X., Yao J. (2024). Advances in morphology-controlled alumina and its supported Pd catalysts: Synthesis and applications. Chem. Soc. Rev..

