# Supplementary material for: Non-Invasive Rayleigh, Raman, and Chromium-Fluorescence Study of Phase Transitions: β-Alumina into γ-Alumina ‘Single’ Crystal and Then to α-Alumina
Source: Materials (Basel). 2025 Oct 12;18(20):4682. doi: 10.3390/ma18204682 (PMC12565712; doi:10.3390/ma18204682)
Supplement: Supplementary file 1 [file materials-18-04682-s001.zip › materials-3898175-supplementary.pdf]

Supplementary Materials

Non-invasive Rayleigh, Raman, and Chromium-fluorescence study of phase transitions: β-alumina into γ-alumina 'single' crystal and then to α-alumina

Juliette Redonnet [1,2], Gulsu Simsek-Franci [3] and Philippe Colomban [4,*]

[1] Centre des Matériaux, Mines Paris PSL, Campus de l'Innovation, Versailles, France ; juliette.redonnet@mines.paris.psi.eu

[2] Saint-Gobain Research Province, Cavaillon, France

[3] Department of Materials Science and Nanotechnology Engineering, Faculty of Engineering, Istanbul Gedik University, Cumhuriyet Mah İlkbahar Sok. No:1 Kartal, 34876 Istanbul, Türkiye; gulsu.simsek@gedik.edu.tr

[4] MONARIS UMR8233, Sorbonne Université, CNRS, Campus P.-et-M. Curie, 4 Place Jussieu, 75005 Paris, France;

* Correspondence: philippe.colomban@sorbonne-universite.fr

850°C indexation

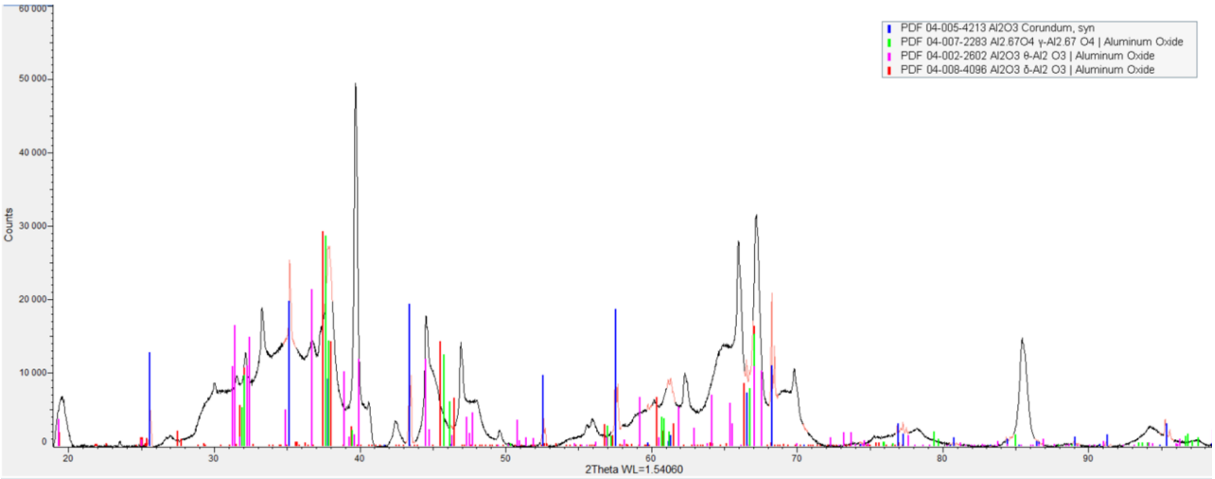

1000°C indexation

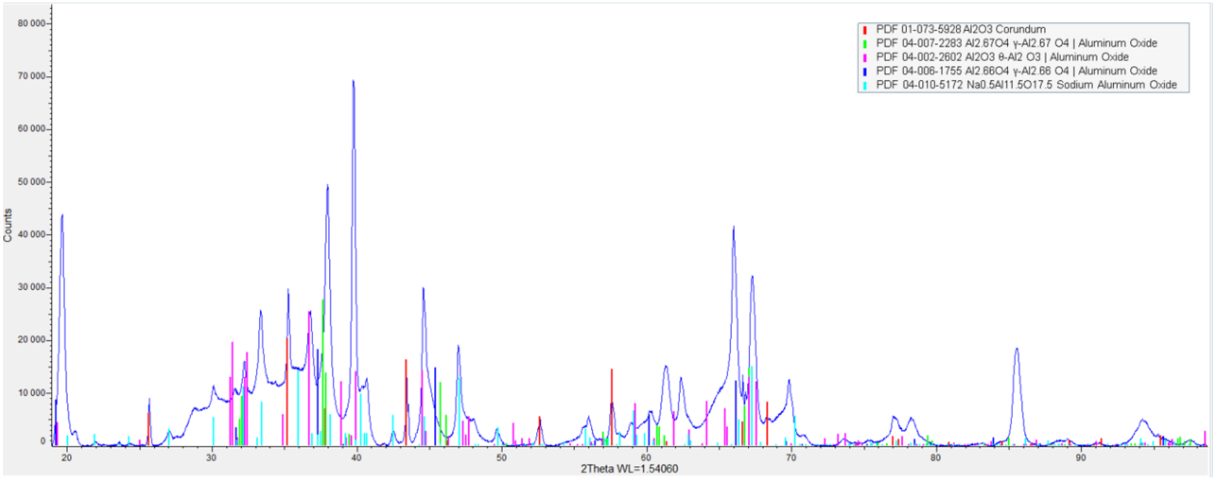

1100°C indexation

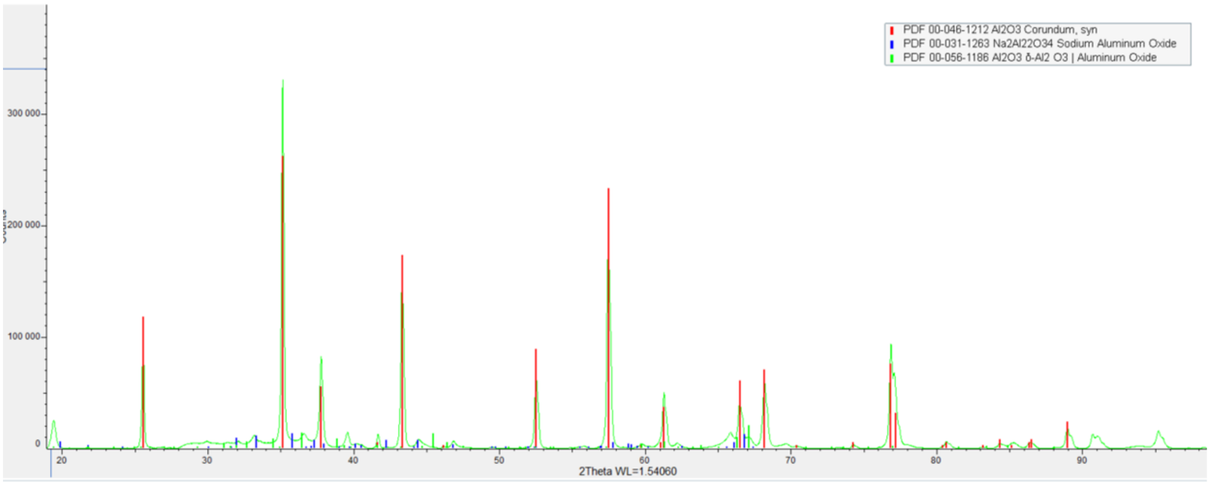

Figure S1. XRD spectra of powdered crystals heated at 850°C, 1000°C and 1100°C
